# Analysis of risk factors for lower limb deep vein thrombosis in patients after Lumbar Fusion Surgery

**DOI:** 10.12669/pjms.37.1.3041

**Published:** 2021

**Authors:** Qiang Li, Zongxue Yu, Xiao Chen, Wenli Zhang

**Affiliations:** 1 Qiang Li, Vascular Surgery Department, Qingdao Hiser Medical Group, Qingdao, Shandong Province, 266033, China; 2 Zongxue Yu, Endocrinology Department, The Third People’s Hospital of Qingdao, Qingdao, Shandong Province, 266041, China; 3 Xiao Chen, Vascular Surgery Department, Qingdao Hiser Medical Group, Qingdao, Shandong Province, 266033, China; 4 Wenli Zhang, Vascular Surgery Department, Qingdao Hiser Medical Group, Qingdao, Shandong Province, 266033, China

**Keywords:** Risk factors, Deep vein thrombosis, Lumbar fusion surgery

## Abstract

**Objective:**

To identify the risk factors of deep vein thrombosis (DVT) of lower limb in patients undergoing lumbar fusion surgeries, to provide a better understanding of DVT and take prophylactic measures.

**Methods:**

This study was carried out in our hospital between January 2016 and February 2020, patients undergoing lumbar fusion surgeries were included and divided into DVT group and non-DVT group, and the medical data including basic demographics, clinical characteristics, operative data, and routine biochemical parameters were collected and analyzed.

**Results:**

In the current study, 390 cases were included, 48 cases were enrolled in DVT group and 342 in non-DVT group. The incidence of DVT was 12.3%. There were significant differences in age, hyperlipemia, hypertension, occupation type, D-dimer level, hospital stay, and postoperative exercises (p<0.05), but no significant differences in gender, smoking status, drinking status, coronary heart disease, diabetes mellitus, fused segments, and operation time (p>0.05) between the two groups. In multivariate analysis, age>50 years, hypertension, D-dimer>500ug/L were independent risk factors, while postoperative exercises were protective factor for DVT.

**Conclusion:**

Those patients undergoing lumbar fusion surgeries should take more prophylactic measures including postoperative exercises if they were elderly, or hypertensive, or have high D-dimer level, in order to decrease the incidence of DVT.

## INTRODUCTION

Lower limb deep vein thrombosis (DVT), one of the common complications in patients suffering from surgeries, can lead to high mortality once pulmonary embolism occurs.^1^ It is estimated that a large number of patients, approximately 200 000 people, die from pulmonary embolism in the world each year.^2^ Consequently, how to prevent the occurrence of DVT in patients after surgeries is highly valued in the field of medicine.

Lumbar fusion surgery is widely used in the treatment of lumbar degenerative disc disease with a satisfying effectiveness. However, the rate of DVT is high in these patients. In a study of 995 patients who suffered from lumbar fusion surgeries, Yang reported that 223 patients were diagnosed with DVT, and the rate was 22.4%.^3^ In the studies conducted by Zhao,^4^ Yi,^5^ and Li,^6^ the rate of lower limb DVT was 11.8%, 15.3%, and 15.8%, respectively. Moreover, DVT of the lower limbs is usually asymptomatic and the sensitivity of routine screening for asymptomatic DVT is low, and in many cases the fatal pulmonary embolism is usually the first manifestation of DVT^1^. AS a result, it is critical to determine the risk factors of lower limb DVT in patients after lumbar fusion surgeries.

Some scholars have focused on the risk factors of DVT related to lumbar fusion surgery. In a study of 710 patients undergoing lumbar fusion, Zhao found advanced age, hypertension, and increased D-dimer level were risk factors for postoperative DVT.^4^ In another study of 216 patients, Yi found the patients with age ≥60, BMI≥24 kg/m^2^, blood transfusion and postoperative bed time ≥5 days were risk factors to lead to DVT of lower extremity.^5^ In Yan’s study, a total of 507 patients, including 386 patients undergoing lumbar fusion surgery, 36 undergoing decompression surgery and 85 internal fixation for lumbar fractures, they concluded only advanced age and paraplegia were high risk factors of DVT.^7^ Other studies also reported different outcomes.^3^ Apparently, these conclusions are still controversial and need more studies to further clarify the issue.

Therefore, we retrospectively reviewed the patients undergoing lumbar fusion surgeries in the spine department of our hospital, and this study was designed and performed to identify the risk factors of lower limb DVT, in order to provide a better understanding of DVT and take prophylactic measures for patients after lumbar fusion surgeries.

## METHODS

This study was carried out in our hospital between January 2016 and February 2020, patients undergoing lumbar fusion surgeries were included for this study. All the patients were inspected via lower extremity ultrasonography preoperatively and postoperatively to determine if DVT were available. The inclusion criteria were: (1) Patients undergoing lumbar fusion surgery; (2) No deep vein thrombosis was found before operation. (3) Postoperatively patients were diagnosed with DVT. Those patients undergoing other surgeries instead of lumbar fusion surgeries, and those who ever took anticoagulant one week before hospital admission were excluded. This study was approved by the institutional ethics board of our hospital (Dated: 20-05-2020).

The medical data of enrolled patients, including basic demographics and clinical characteristics, operative data, and routine biochemical parameters, were collected and recorded. These data included age, sex, hospital stay, operation time, occupation type, blood pressure, coronary heart disease, diabetes mellitus, postoperative exercises, smoking status, drinking status, blood lipid, and D-dimer. At the second day after admission, venous blood samples were collected, and the level of D-dimer and blood lipid was tested for the patients. The diagnostic criteria for hyperlipemia were determined according to 1997 dyslipidemia prevention advice of china, and 2007 Chinese adult dyslipidemia prevention guide standards.^8^ The blood pressure was measured three times at five-minute intervals, and the mean of measurements was recorded for analysis. When systolic BP ≥140 or diastolic BP ≥90mmHg, the patient was diagnosed with hypertension.^9^ The diagnosis of coronary heart disease and diabetes mellitus was also based on the related criteria.^10^,^11^

Statistical analyses were performed based on SPSS software 22.0. Measurement data were expressed as mean and standard deviation, and statistical analyses were carried out by Student t test or Analysis of variance. Categorical data were presented as frequencies, which were analyzed by Chi square test. Logistic analysis was used to determine the relationship between deep vein thrombosis and potential risk factors. P values <0.05 were considered significant.

## RESULTS

Between January 2016 and February 2020, a total of four hundred and eighty-seven patients were treated surgically in the department of spine of our hospital. Among the 487 cases, ninety-seven were excluded because of incomplete clinical data or not meeting the inclusion criteria, and 390 cases were enrolled in this study. Of the 390 cases, 262 were males and 128 females, and their age ranged from 39 to 81 years. After lumbar fusion surgery, 48 cases were diagnosed with DVT and enrolled in DVT group, in which no pulmonary embolism occurred, and the remaining 342 cases were enrolled in non-DVT group.

In the DVT group, DVT was found in left lower extremity, right lower extremity, and both lower extremities in 22, 15, and 11 patients, respectively. As to the affected veins, femoral vein thrombosis was found in two cases, deep femoral vein thrombosis in three cases, superficial femoral vein thrombosis in two cases, popliteal vein thrombosis in six cases, posterior tibial vein thrombosis in six cases, anterior tibial vein thrombosis in one case, calf muscle vein thrombosis in 20 cases, and peroneal vein thrombosis in 19 cases. The incidence of DVT after lumbar fusion surgery was 12.3% in the current study.

**Fig.1:**
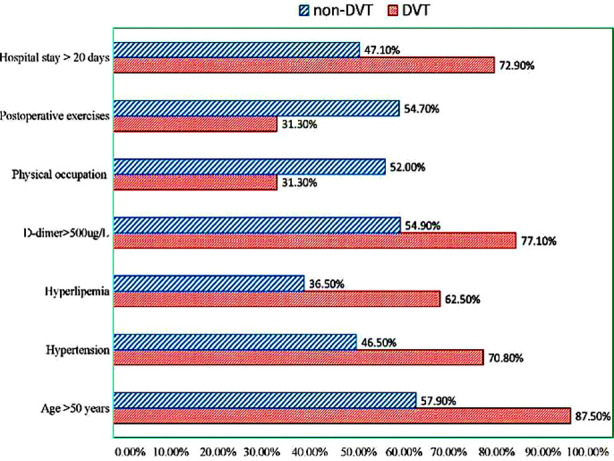
The comparison of clinical data (rate) in univariate analysis between groups.

The comparison of the clinical characteristics between the DVT and non-DVT groups is shown in Table-I. There were significant differences in age, hyperlipemia, hypertension, occupation type, D-dimer level, hospital stay, and postoperative exercises (p<0.05) (Fig.1), but no significant differences in gender, smoking status, drinking status, coronary heart disease, diabetes mellitus, fused segments, and operation time (p>0.05, Table-I) between the two groups.

**Table-I:** The clinical characteristics of the two groups.

*Factors*	*DVT*	*non-DVT*	*P value*

	n=48	n=342	
Age > 50 years (n)			0.0002
yes	42	198	
no	6	144	
Gender (n)			0.807
Male	31	231	
Female	17	111	
Hypertension (n)			0.002
yes	34	159	
no	14	183	
Hyperlipemia (n)			0.001
yes	30	125	
no	18	217	
Coronary heart disease (n)			0.99
yes	30	210	
no	18	132	
Diabetes mellitus (n)			0.26
yes	25	145	
no	23	197	
Level of D-dimer (n)			0.005
>500ug/L	37	188	
<500ug/L	11	154	
Operation time>3 hours (n)			0.95
yes	27	198	
no	21	144	
Fused level (n)			0.706
One level	32	207	
Two levels	15	128	
Three levels or more	1	7	
Occupation (n)			0.01
Physical	15	178	
Mental	33	164	
Postoperative exercises (n)			0.003
yes	15	187	
no	33	155	
Smoking (n)			
yes	29	166	0.16
no	19	176	
Drinking (n)			0.56
yes	24	152	
no	24	190	
Hospital stay> 20 days (n)			0.001
yes	35	161	
no	13	181	

Note:DVT= deep vein thrombosis

In multivariate analysis, age>50 years, hypertension, D-dimer>500ug/L were risk factors, while postoperative exercises were protective factor for DVT after lumbar fusion surgery (Table-II).

**Table-II:** The independent risk factors for deep venous thrombosis.

Factors	P value	OR (95% CI)

Age > 50 years	0.001	4.563(1.893-9.584)
Hypertension	0.003	2.681(1.196-5.372)
D-dimer>500ug/L	0.01	1.439(1.171-3.592)
Postoperative exercises	0.02	0.266(0.183-0.754)

## DISCUSSION

In the current study, a retrospective analysis of 390 postoperative patients in the department of spine of our hospital was performed to determine the risk factors of DVT after lumbar fusion surgeries. This study may help surgeons take measures for patients in preventing the occurrence of DVT.

Some studies have reported that hypertension and D-dimer have close correlations with DVT. In Zhao’s study, both D-dimer and hypertension were identified to be significantly associated with DVT.^4^ In addition, in a study of 1179 patients by Liu and colleagues, a similar viewpoint was confirmed.^12^ In the present study, we had the same conclusion, and found both factors are risk factors of DVT after lumbar fusion surgeries, further confirming the conclusion of previous literature. D-dimer, as a degradation product of cross-linked fibrin, work as an indicator of both coagulation- and fibrinolysis activation and an elevated D-dimer level is associated with an increased risk in thrombin formation.^13^ In addition, some studies have demonstrated the correlation between D-dimer and blood pressure. A study advocated that the level of D-dimer was positively correlated with gestational hypertension,^14^ and another study found a positive association between blood pressure and elevated D-dimer in black South Africans.^13^ In the present study, as both D-dimer and hypertension are risk factors of DVT, we believe there may be a positive association between two factors in Chinese population.

In this study, we found age > 50 years was an independent risk factor of DVT in patients after lumbar fusion surgeries, indicating patients older than 50 years are more liable to suffer from DVT. Elderly patients are closely associated with the occurrence of DVT, the reasons may be contributed to vascular sclerosis, high blood viscosity as well as poor venous valve function^1^. In addition, most of old patients may have prolonged high blood pressure, which may damages the vessel endothelium,^4^ and lumbar fusion surgeries may usually cause reduced activities in elderly patients,^1^,^4^ all of which also contributes to the formation of DVT. In Zhao, Yi, and Yan’s study, they also advocated that advanced age was an independent risk factor for DVT after lumbar fusion surgeries.^4^,^7^,^15^

Based on the univariate analysis, we found the rate of postoperative exercises were significantly higher in non-DVT group than DVT group. In multivariate analysis, it was an independently protective factor of DVT, demonstrating postoperative exercises may be helpful in decreasing the incidence of DVT. In one of our previous studies including 376 patients after neurosurgeries, we concluded the same conclusion.^1^ Although the patients were treated using different surgeries, the mechanism of DVT may be similar. In another study of 315 patients treated in neurospine intensive care unit and who received foot exercises as a method of DVT prevention, Palamone found those who developed DVT had a significantly lower compliance rate with the ROM exercises than did those who did not develop DVT.^16^ Scholars found that enhanced popliteal blood velocity and volume flow are critical factors in preventing venous stasis and DVT, and simple toe and ankle exercises can maintain venous return in patients with below-knee cast immobilization.^17^ Subsequently, these previously published studies together with the current study indicate that postoperative exercises play an important role in preventing DVT.

In summary, we found in the current study that advanced age, higher level of D-dimer, and hypertension were risk factors, but postoperative exercises were protective factor of DVT, indicating those patients undergoing lumbar fusion surgeries should take more prophylactic measures including postoperative exercises if they were elderly, or hypertensive, or have high D-dimer level, in order to decrease the incidence of DVT.

### Limitations of the study

First, in this study we evaluated the correlations between the factors such as blood pressure, serum lipid, drinking and smoking status and the occurrence of DVT, but some other associated factors such as fibrinogen, body mass index and anticoagulant drugs were not included, which may affect the final conclusion. Second, the current study was performed retrospectively instead of prospectively, a prospectively conducted study may be better in clarifying the risk factors. Third, both D-dimer and hypertension are risk factors of DVT, indicating there may have a positive association between two factors in Chinese population, but we didn’t study the correlation in the current study. Hence, more studies should be performed in the future.

### Authors’ Contribution:

**WLZ** conceived, designed the project and performed the statistical analysis,

**QL, ZXY, and XC** did data collection and prepared the manuscript,

**QL and WLZ** did review and final approval of the manuscript.

**WLZ** is responsible for the accuracy and integrity of the work.
